# A Case of Softening of the Brain, with Strumous Tubercles, Causing Apoplexy and Palsy

**Published:** 1833-10

**Authors:** C. J. Roberts

**Affiliations:** one of the late Physicians to the General Dispensary, and Physician to the Infant Orphan Asylum.


### *A Case of Softening of the Brain, with Strumous Tubercles, causing Apoplexy and Palsy.* By C. J. Roberts, M.D. one of the late Physicians to the General Dispensary, and Physician to the Infant Orphan Asylum.

Acute inflammation of the tunica arachnoidea and pia mater, terminating by effusion into the cerebral cavities, calls for little or no comment, for, generally speaking, the practice to be pursued is so obvious as hardly to leave room for doubt; for the patient sinks, unless the disease is promptly met by active practice; but it is when an inflammation of a chronic character is already about to terminate by effusion of serum into the ventricles, and symptoms of pressure are becoming apparent, that the inexperienced practitioner first sees the perilous situation of his patient. In many cases, the approach of the disease is so insidious, as to completely throw the practitioner off his guard, and it is not until it is past remedy that he is aware of the danger. This is more peculiarly applicable to that disease to which Dr. Abercrombie of Edinburgh has given the title of "Chronic Inflammation of the Brain," and which most commonly terminates in that peculiar alteration in the structure of the cerebral mass which has been termed by the French writers "ramollissement," and by us, softening of the brain.

The symptoms, as detailed by both Dr. Abercrombie and the French authors, on the subject, are so obscure and passive, as rarely to arouse either the patient or the practitioner to a due sense of their dangerous tendency, unless the latter has been previously acquainted with attacks of this description, when he will be on his guard against their insidiousness and peril. In the case which is the subject of this paper, the disease did not commence its aggression in its usual way, but shewed itself in another guise, putting on the appearance in the first instance of an apoplectic seizure, and continuing under that of palsy, and from occurring in so young a patient, its progress was rendered more obscure.

Thomas Garrett, æt. four. Admitted a patient of the General Dispensary, November 29, 1832.

On the 26th of November, while at play, in the afternoon, he was suddenly seized with giddiness and fell down, striking his left temple with so much violence as to bruise it considerably. On being lifted up he was found to be insensible, and remained in that state for ten minutes. On recovering, his speech was found to be imperfect, and which remains indistinct: and he also partially lost the power of controlling his left hand, but not his left leg. At present he is very drowsy, particularly in the day time; and during the night sleeps very heavily, snoring deeply; complains of headach. He is thirsty, has no appetite; his bowels are regular. His mother has observed that, since the attack, his sensorial powers are much impaired, that he has become mischievous, and that during play he will carefully keep in the room, and avoid going near to the stairs, as if he was fearful of losing his balance and falling down them: she adds, that he had complained of headach for about a fortnight previous to the sudden loss of speech and palsy of the left arm. He was bled with leeches; cold lotions were applied to his head, and he was purged. This treatment was followed by considerable relief, and he nearly recovered the use of his arm and his speech, but the latter was still tremulous, and he was unwilling to talk. The headach was reported to be gone. This improved state of things continued from the 3d until the 27th of December, when, although not complaining of headach, he was described as being less capable of standing, and to have exhibited symptoms of great irritability of temper. On the 1st of January, the pain in the occiput returned, and he lost the use of the right leg, and dragged it after him; the pupils now became very much dilated. Leeches were again applied with relief, but still the power over both the legs was evidently diminished, and he was unable to stand. He was now put under a mercurial course, in addition to the use of cold lotions and leeching. After using the mercury twice a day for a fortnight his mouth became sore, and he then recovered his mental faculties, and the power to a certain degree over the lower extremities. When the effect of the mercury began to go off, strabismus came on, and the symptoms continued to vary in violence until the 5th of March, when these being materially increased, it was thought advisable, at a consultation which was held upon his case by the physicians of the dispensary, that the head should be relieved by bleeding from the jugular vein. His father objected to this, and he was afterwards cupped to the extent of ℥vi. and with very great relief. He continued to linger in this paralytic state until the 15th of April, when he died. He was at times free from pain in the head, and at others he was afflicted with it severely.

We were allowed to examine the body: on removing the skullcap, which was preternaturally thin, the dura mater was observed to be more vascular than usual. The tunica arachnoides was thickened, and was also adhering in several places to the dura mater. The vessels of the pia mater were much gorged, and the entire cerebral mass had lost its usual firmness, was of the consistence of custard, and was spotted all over with bloody points. The lateral ventricles were much dilated, and contained nearly ℥viij. of fluid. The thalami nervorum opticorum were much enlarged, and in the centre of the left one, a cluster of yellow strumous tubercles were found, some of them approaching in size a large nut, or small walnut. The vessels of the cerebellum were very much gorged; the tuber annulare and crura cerebelli were harder than natural, and in the left crus a cluster of similar tubercles was found. There were also two tubercles about the size of horse-beans on the middle lobe of the brain.

The other cavities presented no particular appearance.

It was not supposed, when he first came to the dispensary, that any organic destruction of the cerebral mass existed, but that being a child of a gross habit of body, he had some determination to the brain, which would be relieved by depletion.

The softening of the brain, although it must have proceeded to some extent when first brought under my care never manifested itself in its usual way; and the presence of strumous tubercles in the brain was never once suspected, as the symptoms usually denoting them were never well marked.

The alteration which takes place in the structure of the brain after attacks of this description is very peculiar, for although, so long as the mass is kept in situ by the membranes, the form is unaltered, no sooner is this support removed than the entire cerebrum is found to have lost its usual firmness, and to be reduced to a pulpy consistence. It is not surprising that such an attack should be followed by so great a disorganization: but it is very remarkable that so important an organ as the brain should suffer so complete and destructive a change, unattended by any symptoms which shall decidedly shew the serious mischief which is so rapidly proceeding. The cerebral functions appear to be still performed, although laboriously and imperfectly, but yet not to that extent of incapacity and invalidation which is witnessed when pressure only is the active cause. We very often find tumours in the brain producing local pressure, but not exciting those symptoms which are usually looked upon as denoting its presence, but setting up other irritations, which, although less permanent in their violence, are no less durable in their attendance, as we find in old cases of epilepsy, arising from these bodies. In the case just detailed it is a fair conclusion, I conceive, to believe that the tumours were the cause of the paralytic symptoms, and that, instead of giving origin to epileptic paroxysms, they caused pressure, and gave rise to a genuine attack of palsy. The mode of aggression was decidedly similar to the apoplexy of maturer years, and that the symptoms, although relieved by the usual antiphlogistic treatment, did not entirely give way. The paralysis also was perfect, and as the disease advanced paraplegia supervened. After every bleeding he was certainly better, and when the mouth became affected by the mercury the signs of improvement were more decisive; these, however, were but temporary, and he gradually, and insensibly sank.

In most of the cases mentioned by Dr. Abercrombie, of softening of the brain in which tumours were discovered, he says, that in nearly all, the symptoms were accompanied by convulsions, and in others by chorea, but in this no convulsions were observed; the attack was completely apoplectic, and was succeeded by the consequence of apoplexy—complete palsy.

Wepfer, in his "Historia Apoplecticorum," relates concisely two cases of adults who were seized in a like manner, and in whose brains similar tumours were found.

